# ﻿Taxonomic and nomenclatural notes on *Geodiaprialongiceps* Kieffer, 1911 (Hymenoptera, Diapriidae) and synonymy of the genus *Geodiapria* Kieffer, 1910

**DOI:** 10.3897/zookeys.1183.110952

**Published:** 2023-10-23

**Authors:** Jeremy Hübner, Vasilisa G. Chemyreva, David G. Notton

**Affiliations:** 1 Zoologische Staatssammlung München, Muenchhausenstr. 21, 81247 Munich, Germany Zoologische Staatssammlung München Munich Germany; 2 Zoological Institute, Russian Academy of Sciences, 1 Universitetskaya Emb., St. Petersburg 199034, Russia Zoological Institute, Russian Academy of Sciences St. Petersburg Russia; 3 Department of Natural Sciences, National Museums Collection Centre, 242 West Granton Road, Granton, Edinburgh EH5 1JA, UK National Museums Collection Centre Edinburgh United Kingdom

**Keywords:** *Basalysrufocinctus*, DNA-barcoding, first record, integrative taxonomy, parasitoid wasp, species concepts

## Abstract

This paper reviews the status of *Geodiapria* and its nominotypical and only included species *G.longiceps*. *Geodiapria* was previously understood to be very similar to, and doubtfully separated from the genus *Basalys*. We use integrative taxonomy (morphology, DNA-barcoding, phylogenetic tree building) to show that the valid name for what was *G.longiceps* Kieffer, 1911 is now *Basalysrufocinctus* (Kiefer, 1911) and that *Geodiapria* is consequently a junior synonym of *Basalys***syn. nov.** The following taxa are new synonyms of *B.rufocinctus*: *Loxotropalongiceps* Wasmann, 1909, **syn. nov.**, *G.longiceps* Kieffer, 1911, **syn. nov.**, *L.rufosignata* Kieffer, 1911, **syn. nov**. *Basalysrufocinctus* is newly reported from Corsica, Germany, Norway and Spain.

## ﻿Introduction

Parasitoid wasps of the family Diapriidae are speciose and distributed worldwide, and while about 50% of its diversity is estimated to be unknown to science, there are few experts working on this family. Small size (c. 1–4 mm), wide distribution, cryptic diversity, sexual dimorphism, and previous poor taxonomy and lack of critical study of types are some of the problems researchers face when dealing with Diapriidae. The taxonomy of this group still therefore presents many interesting challenges. The status of the genus *Geodiapria* and its single included species *G.longiceps* Kieffer, 1911 has been a taxonomic problem for some time because of its close relation to *Basalys*, in particular species such as *B.rufocinctus* (Kiefer, 1911) with similar distinctive reddish flattened petiolar hairs. The question this paper seeks to resolve is whether or not *Geodiapria* is valid. *Geodiapria* was first described in a key by Kieffer (1910) who separated it from *Loxotropa* auctt. (now *Basalys* in part) and *Basalys* sensu stricto simply by the lack of a basal vein, adding later (Kieffer 1911a) that the form of the head, longer than wide and a little wider in front than behind, was also distinctive. It was clearly similar to *Basalys* because Kieffer had previously considered the same material to be a *Loxotropa* auctt. (Wasmann, 1909). Kieffer (1911b) then described two species of *Loxotropa* auctt. with the same distinctive reddish flattened petiolar hairs: *L.rufosignata* said to have a head slightly longer than wide and reduced wings without distinct veins; and *L.rufocincta* with an almost square head and with an almost hyaline basal vein. Pschorn-Walcher (1957) examined the type of *G.longiceps* and considered *Geodiapria* to be very close to *Loxotropa* auctt., noting that the absence of the basal vein could be a consequence of wing reduction, but did not make a decision on the validity of *Geodiapria* because of lack of material. Since more material is now available, it is timely to reexamine the question of the validity of *Geodiapria* using an integrative approach combining morphotaxonomy and DNA barcoding (Ratnasingham and Hebert 2007). We examined 18 examples including types of four relevant nominal species, including *L.rufosignata* and *L.rufocincta*, and provide an up to date nomenclatural summary, presenting the first genetic results, including the DNA-barcode placing *Geodiapria* in its proper context.

## ﻿Material and methods

The specimens of *B.rufocinctus* used for the CO1 DNA barcoding were collected in July 2021 in the Dammbach Valley (Spessart Nature Park) on an orchard meadow, using a Malaise trap. The sequencing was conducted at Canadian Centre for DNA Barcoding (Guelph, Canada) using a voucher recovery protocol. Tree building was undertaken using IQ TREE (server version 1.6.12, Trifinopoulos et al. 2016) using the default settings with 1000 generations. MODELFINDER determined GTR+F+I+G4 to be the best fitting substitution model. The resulting tree was edited using FIGTREE v. 1.4.4 (Rambaut 2010) and INKSCAPE v. 1.1 (https://inkscape.org/de/).

**Repository acronyms**:

**DNPC** David Notton personal collection, United Kingdom

**MCSN** Museo Civico di Storia Naturale “Giacomo Doria”, Genoa, Italy

**MNHN** Muséum national d’Histoire naturelle, Paris, France

**NHME** Natural History Museum, Maastricht, Netherlands

**NHMUK** Natural History Museum, London, United Kingdom

**SNSB-ZSM** Bavarian State Collection, Munich, Germany

## ﻿Taxonomy

### ﻿ Basalys

Taxon classificationAnimaliaHymenopteraDiapriidae

Westwood, 1833

4981AF53-0749-57FF-B7A0-6A030C654546

Basalys Westwood, 1833: 343. Type species Basalysfumipennis Westwood, 1833 by monotypy.
Loxotropa auctt. nec Förster, 1856.
Geodiapria Kieffer, 1910: 707, syn. nov. Type species G.longiceps Kieffer, 1911 by subsequent monotypy.

#### Notes.

Other generic synonyms are omitted from the above list for simplicity. A diagnosis and detailed description of *Basalys* was given by Masner and García (2002), hence, only a brief diagnosis is given here. Further information on synonyms can be obtained from Johnson (1992).

#### Diagnosis.

Small, smooth and shining wasps; head and mesosoma with long scattered hairs; antennal shelf usually distinctly prominent; female antenna 12-segmented, with strongly abrupt 3- or 4-segmented clava; male antenna 14-segmented with A4 distinctly modified; fore wing with submarginal vein slightly remote from fore margin of wing, costal vein absent, stigmal vein often moderately developed, basal vein always present in macropterous forms, straight, usually strongly pigmented, perpendicular to but never contiguous with submarginal vein.

#### Remarks.

We discovered that the type species of *Geodiapria*, that is *G.longiceps*, is a *Basalys*, a synonym of *B.rufocinctus* (see below) and so *Geodiapria* becomes a junior synonym of *Basalys* syn. nov.

### ﻿ Basalys rufocinctus

Taxon classificationAnimaliaHymenopteraDiapriidae

(Kieffer, 1911)

A29A6007-9589-533D-B6DB-94A521E99F45

Loxotropa longiceps Wasmann, 1909: 68, 172, syn. nov., preoccupied nec B.longiceps (Ashmead, 1893).
Geodiapria longiceps Kieffer, 1911a: 897, syn. nov., preoccupied nec B.longiceps (Ashmead, 1893).
Loxotroparufocinсta Kieffer, 1911b: 916, 939 takes precedence over L.rufosignata by first revisor action.
Loxotropa rufosignata Kieffer, 1911b: 914, syn. nov.

#### BIN number.

BOLD_BIN: AEW6196 (Ratnasingham and Hebert 2007).

#### Type material.

***Holotype*** ♀ of *Loxotropalongiceps* labelled: “Allotype ♂ (!)/ Solenopsiaimitatrix/ Wasmann, err. det.!; Holotype ♀/ Geodiaprialongiceps/ Kieffer, 1911; Loxotropa/ longiceps n. sp./ ♀ Kieff.; 5.98. Exaet./ b. Solenopsis; Solenopsis m/ Kol. 293. sang [=colony #293 of *Formicasanguinea*].” (NHME) (Fig. 2). ***Holotype*** ♀ of *Geodiaprialongiceps* - the same specimen as the holotype of *Loxotropalongiceps* q.v. ***Holotype*** ♀ of *Loxotroparufosignata* labelled: “Is. Giglio/ IV.1902/ G. Doria; Loxotropa/ rufosignata; ♀” (MCSN) (Fig. 3). ***Syntypes*** 2♀ 3♂ of *Loxotropa rufocinсta*: 2♀ labelled: “Holotype [sic – there is no original designation]; Bitche; Loxotropa/ rufocincta; Muséum Paris/ 1957/ coll. Kieffer. 2♂ labelled: Loxotropa/ rufocincta; Bitche; ♂; Allotype; Muséum Paris/ 1957/ coll. Kieffer. ♂ labelled: Paratype; Muséum Paris/ 1957/ coll. Kieffer; Bitche” (MNHN).

#### Other material.

**Denmark** • ♀; N. E. Zealand, Tisvilde Hegn; 56°02'N, 12°04'E; 4 May 1994; P.N. Buhl leg. (DNPC). **France** • ♂; Corsica, Corse du Sud, Bastelicaccia nr. Ajaccio;41°55'N, 08°30'E; 14–21 Jun. 1996; C. Villemant leg.; Malaise trap, *Quercussuber* stand (DNPC) • ♀; Gard, Mont Ventoux, Malaucène; 44°13'N, 05°08'E; 1–8 Jul. 1997; C. Villemant leg.; maquis, *Quercusilex* (DNPC) • ♂; same locality; 5–12 Aug. 1997; C. Villemant leg.; maquis, *Quercusilex* (DNPC). **Germany** • ♀; Bavaria, Dammbach, Dammbachtal; 49°51′58″N, 09°19′30″E; 338 m a.s.l.; 16 Jul. 2021; J. Hübner leg.; nutrient poor grassland; ZSM-HYM-42434-GO2 (BOLDSYSTEMS Process ID: DTIII5299-22; GenBank accession ID: OR450821) (SNSB-ZSM) • ♀ same locality; 16 Jul. 2021; J. Hübner leg.; nutrient poor grassland; ZSM-HYM-42433-H11 (BOLDSYSTEMS Process ID: DTIII5225-22; GenBank accession ID: OR450822) (DNPC). **Norway** • ♀; Onsøy, Hankø Bloksberg, EIS 20, Ø; 3–29 Jun. 1995; O. Hanssen & J.I.I. Båtvik leg.;pitfall trap (DNPC). **Spain** • ♀; Granada, Calahonda; Jul. 1987; L. Lockey leg.; Malaise trap, (DNPC) • ♀; Granada, Sierra Nevada; 1600 m a.s.l.; 10 Apr. 1959; C. Besuchet leg. (NHMUK). **United Kingdom** • ♀; Cheshire, Abbotts Moss; 53°12′27″N, 02°36′23″W; 12 Oct. 1990; D.G. Notton leg.; swept, stream (DNPC) • 3♀; Norfolk, Santon Downham; 52°27′45″N, 00°40′29″E; 15 Aug. 1984; J. Field leg.; Malaise trap, heath with *Betula* and *Pinus* (DNPC) • 1♂; same locality; 18–25 Aug. 1983; J. Field leg. (DNPC).

#### Diagnosis.

**Figure 1.**
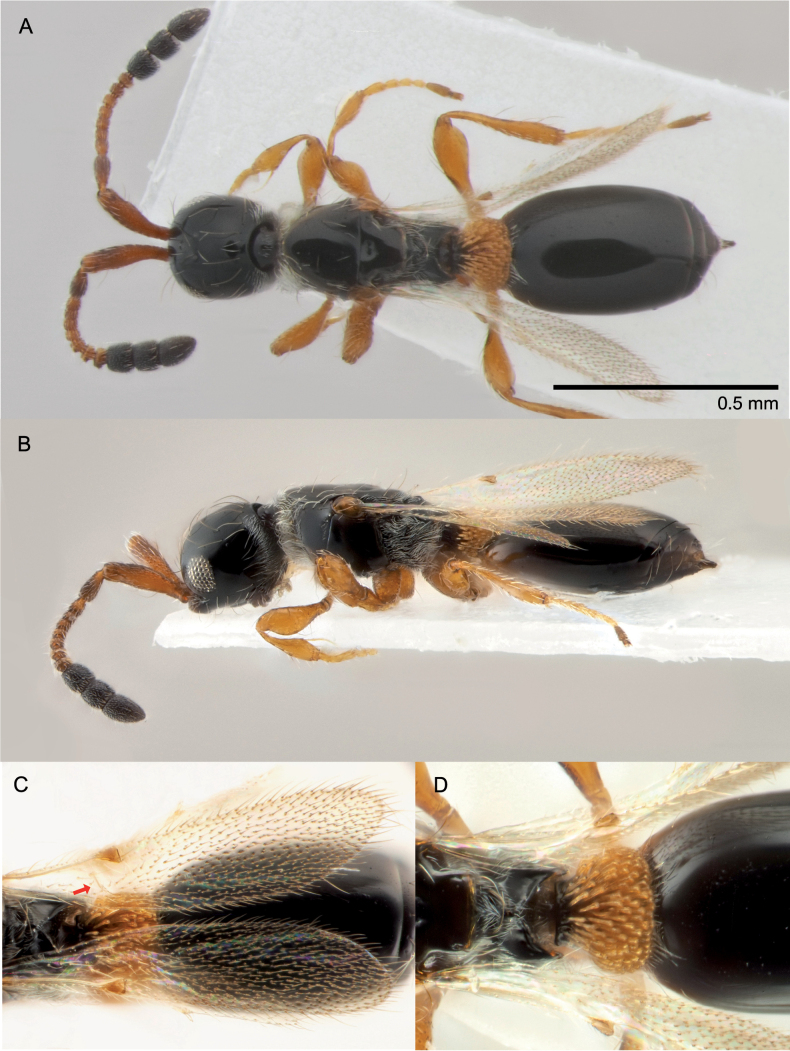
*Basalysrufocinctus* (Kieffer, 1911) ♀: **A** habitus, dorsal view **B** habitus, lateral view **C** wing with reduced venation (arrow) **D** close-up of petiole.

***Female*** Head elongate, rounded, about 1.2 times as long as wide; frons without angles or teeth; antenna 12-segmented with abrupt 3-segmented clava; A11 transverse in lateral view, as long as wide in dorsal view; A6–A9 transverse in lateral view (Fig. 1A); mesonotum and scutellum slightly convex in longer winged individuals, almost flat in shorter winged individuals (Fig. 1B), anterior pronotum with a ruff of whitish setae; anterior scutellar pit small and transverse, less than one third the width of the scutellum; propodeum with medial keel slightly raised anteriorly, less so in short winged individuals; fore wing variable in length, at most extending well beyond apex of gaster, at least reaching anterior margin of petiole; basal vein present in longer winged individuals although hard to see as it is fine and barely pigmented, absent in shorter winged individuals; femora of all legs broadened medially, fore femora 2.2–2.3 times as long as wide in lateral view, with sharp keel ventrally; petiole densely covered dorsally and laterally with long orange flattened setae (Fig. 1D); basal margin of large tergite with two whitish hair tufts more or less concealed under petiolar setae; disc of large tergite normally bare, although the shortest winged individuals, e.g. the type of *L.rufosignata*, may have some long setae. ***Male*** As for female except antenna 14-segmented with A4 expanded posteriorly subtriangular with a fine flange; A5 elongate, flagellar segments becoming shorter towards apex, A13 more or less quadrate; fore wing variable in length at least reaching apex of gaster, at most extending well beyond it; basal vein present, fine, barely pigmented; femora slightly less broadened than female. Body length 1.3–2.2 mm (♀); 1.5–2.4 mm (♂).

**Figure 2.**
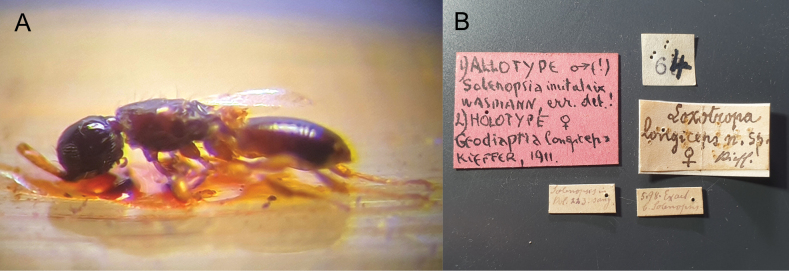
Holotype ♀ of *Loxotropalongiceps* (Wasmann, 1909), the same specimen is also the holotype ♀ of *Geodiaprialongiceps* Kieffer, 1911: **A** habitus, lateral view **B** labels.

**Figure 3.**
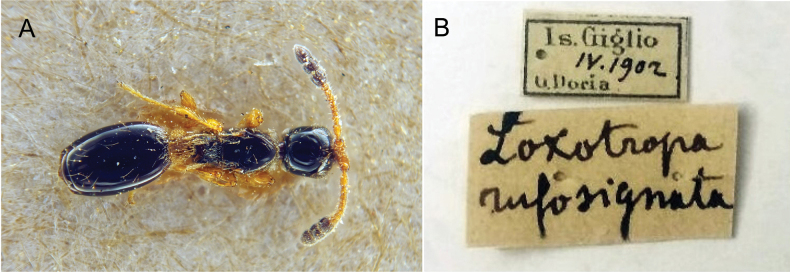
Holotype ♀ of *Loxotroparufosignata* Kieffer, 1911: **A** habitus, dorsal view **B** labels.

#### Distribution.

Czechia (Macek 1989 as *B.rufocincta* [sic]); Denmark (Buhl 1998 as *B.rufocincta* [sic]) confirmed here; France - mainland France (Kieffer 1911b as *L.rufocincta*) confirmed here; France - Corsica (new record); Germany (new record); Italy (Kieffer 1911b as *L.rufosignata*); Netherlands (Wasmann 1909 as *L.longiceps*); Norway (new record); Spain (new record); Sweden (Hedqvist 2007 as *B.ruficincta* [sic]); United Kingdom (Nixon 1980 as *B.rufocincta* [sic]) confirmed here.

#### Biology.

Host unknown. *Basalysrufocinctus* has previously been considered to be a myrmecophile but the evidence is weak. Of all the specimens we have seen only one, Wasmann’s, was found in an ant nest, in a mixed colony of *Solenopsisfugax* and *Formicasanguinea*, and may have entered the nest by accident. Wasmann provided no ethological observations to demonstrate myrmecophily and the species has no obvious morphological adaptation for myrmecophily when compared to other *Basalys*.

#### Remarks.

From the extensive material examined we recognised only one taxon, diagnosed above, and with more variation than previously understood. Most importantly we found that the head was always elongate when seen from above, also significant variation in fore wing length, and expression of the basal vein which was present and weakly pigmented in longer winged individuals, becoming hyaline and then altogether absent in shorter winged individuals. This taxon is therefore a *Basalys* since there is no significant morphological difference: some other species of *Basalys* are known to have elongate heads, also some other *Basalys* have the basal vein absent in short-winged individuals. Based on our examination of the type specimens we consider all four nominal species above, including *Geodiaprialongiceps*, belong to this taxon.

Further support for the generic placement of *B.rufocinctus* is based on genetic analyses. A representative ML tree (Appendix 1; *Idiotypamaritima* (Haliday, 1833) as outgroup, 1000 generations) with 76 Diapriini specimens shows *B.rufocinctus* nested within a *Basalys* clade (Appendix 1). The obtained sequences are publicly available on the BOLDSYSTEMS platform (Ratnasingham and Hebert 2007).

Some nomenclatural notes are necessary:

1. We differ from some authors in recognising *Loxotropalongiceps* as a nominal species separate from, and not just a combination of, *Geodiaprialongiceps*. *Loxotropalongiceps* is available from Wasmann’s (1909) paper where the name is first used. The name is made available by indication (ICZN 1999: Code art. 12.2.1) since Wasmann refers to his description (Wasmann, 1899) of a specimen previously misidentified as a male of *Solenopsiaimitatrix* Wasmann, 1899. Although Wasmann attributes the name to Kieffer, the author of the name is actually Wasmann because he was responsible for publishing the name and writing the prior description (ICZN 1999: Code art. 50.1). The oldest available name for the taxon is thus *L.longiceps* Wasmann, 1909.
2. As *L.longiceps* is transferred to *Basalys* it becomes a secondary junior homonym of *B.longiceps* (Ashmead, 1893) so is invalid.
3. The next oldest available name is *G.longiceps* described as new by Kieffer (1911a). The date of publication is early 1911: evidence comes from the NHMUK copy which has a library stamp 25 Feb. 1911, and the page bound into the end of vol. 10 of *Species des Hyménoptères d’Europe et d’Algérie* which says 1 Mar. 1911.
4. As *G.longiceps* is transferred to *Basalys* it becomes a secondary junior homonym of *B.longiceps* (Ashmead, 1893) so is invalid.
5. The next oldest available names are *L.rufosignata* Kieffer, 1911b and *L.rufocincta* Kieffer, 1911b which were published simultaneously in mid-1911: the page bound into the end of vol. 10 of *Species des Hyménoptères d’Europe et d’Algérie* says 1 Jun. 1911.
6. Since the only two remaining potentially valid names are published simultaneously, we here make a first revisor action to determine precedence thus: *L.rufocincta* has precedence over *L.rufosignata*. We have chosen *L.rufocincta* because this is the more widely used name.
7. *L.longiceps*, *G.longiceps* and *L.rufosignata* are all new synonyms of *L.rufocincta*.
8. The valid name is thus *Basalysrufocinctus*, a combination first recognised by Nixon (1980).
9. Despite previous misspellings, when in combination with *Basalys*, the correct spelling of the species epithet is *rufocinctus*; the gender of *Basalys* is masculine (Notton (2014).

## Supplementary Material

XML Treatment for Basalys

XML Treatment for Basalys rufocinctus

## Figures and Tables

**Figure A1.**
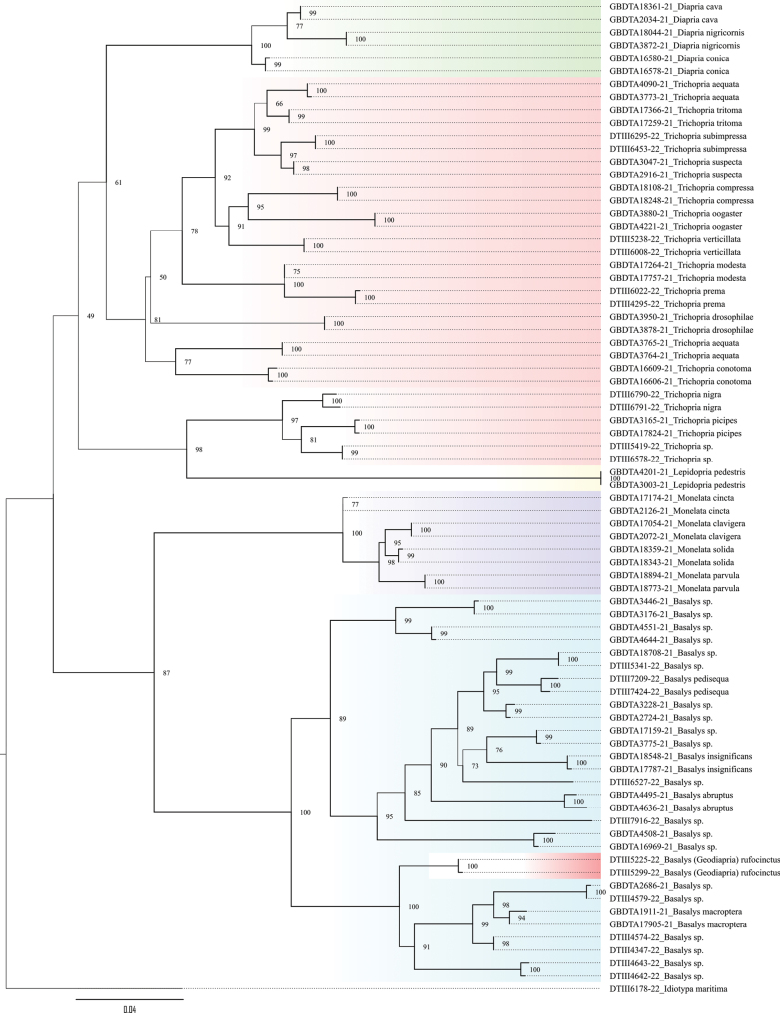
Maximum-likelihood tree of 76 Diapriini specimens, with *Idiotypamaritima* as outgroup. The different genera are color-coded, the numbers on the nodes represent the bootstrap values. Files are openly accessible online at TREEBASE (Piel et al. 2009; http://purl.org/phylo/treebase/phylows/study/TB2:S30685).

## ﻿Additional information

### Author ORCIDs

Jeremy Hübner https://orcid.org/0009-0007-5624-8573

Vasilisa G. Chemyreva https://orcid.org/0000-0002-6547-6259

David G. Notton https://orcid.org/0000-0002-8933-7915

### Data availability

All of the data that support the findings of this study are available in the main text or Supplementary Information.

## Supplementary materials

Supplementary material 1 Supplementary data Data type xlsx https://binary.pensoft.net/file/925549This dataset is made available under the Open Database License (http://opendatacommons.org/licenses/odbl/1.0/). The Open Database License (ODbL) is a license agreement intended to allow users to freely share, modify, and use this Dataset while maintaining this same freedom for others, provided that the original source and author(s) are credited.Jeremy Hübner, Vasilisa G. Chemyreva, David G. Notton
